# Using the Microwell-mesh to culture microtissues in vitro and as a carrier to implant microtissues in vivo into mice

**DOI:** 10.1038/s41598-021-84154-4

**Published:** 2021-03-04

**Authors:** Melissa E. Monterosso, Kathryn Futrega, William B. Lott, Ian Vela, Elizabeth D. Williams, Michael R. Doran

**Affiliations:** 1 School of Biomedical Sciences, Faculty of Health, Queensland University of Technology, Brisbane, Australia; 2 Translational Research Institute, Brisbane, Australia; 3 Centre for Biomedical Technologies (CBT), School of Mechanical, Medical, and Process Engineering (MMPE), Science and Engineering Faculty (SEF), Queensland University of Technology, Brisbane, Australia; 4 Australian Prostate Cancer Research Centre – Queensland (APCRC-Q) and Queensland Bladder Cancer initiative (QBCI), Brisbane, Australia; 5 Department of Urology, Princess Alexandra Hospital, Brisbane, Australia; 6 Mater Research Institute – University of Queensland (UQ), Translational Research Institute (TRI), Brisbane, Australia

**Keywords:** Cancer, Medical research, Biomedical engineering

## Abstract

Prostate cancer (PCa) patient-derived xenografts (PDXs) are commonly propagated by serial transplantation of “pieces” of tumour in mice, but the cellular composition of pieces is not standardised. Herein, we optimised a microwell platform, the Microwell*-*mesh, to aggregate precise numbers of cells into arrays of microtissues, and then implanted the Microwell-mesh into NOD-scid IL2γ^−/−^ (NSG) mice to study microtissue growth. First, mesh pore size was optimised using microtissues assembled from bone marrow-derived stromal cells, with mesh opening dimensions of 100×100 μm achieving superior microtissue vascularisation relative to mesh with 36×36 μm mesh openings. The optimised Microwell-mesh was used to assemble and implant PCa cell microtissue arrays (hereafter microtissues formed from cancer cells are referred to as microtumours) into mice. PCa cells were enriched from three different PDX lines, LuCaP35, LuCaP141, and BM18. 3D microtumours showed greater in vitro viability than 2D cultures, but neither proliferated. Microtumours were successfully established in mice 81% (57 of 70), 67% (4 of 6), 76% (19 of 25) for LuCaP35, LuCaP141, and BM18 PCa cells, respectively. Microtumour growth was tracked using live animal imaging for size or bioluminescence signal. If augmented with further imaging advances and cell bar coding, this microtumour model could enable greater resolution of PCa PDX drug response, and lead to the more efficient use of animals. The concept of microtissue assembly in the Microwell-mesh, and implantation in vivo may also have utility in implantation of islets, hair follicles or other organ-specific cells that self-assemble into 3D structures, providing an important bridge between in vitro assembly of mini-organs and in vivo implantation.

## Introduction

Most prostate cancer (PCa) research is performed using cultured cancer cell lines. While easy to manipulate in culture, cell lines often do not respond to drugs in the same manner as tumours from which the cell lines were originally derived^1,2^. This limitation motivates the use of patient-derived xenografts (PDX), which are only propogated in vivo, and which better mimic the behaviour of the original tumour ^3^. The capacity to establish PDXs has increased markedly with the development of more advanced immune-compromised mouse strains, which make xenograft establishment easier ^4^. PCa PDX establishment generally involves the implantation of “*pieces*” of the original tumour into an immune-compromised animal^5,6^, typically subcutaneously or under the highly vascularised kidney capsule^7,8^. Subsequent propagation of the PDX is generally achieved by transplanting minced “*pieces*” of the original PDX into the next group of recipient mice. A weakness with this approach is that the composition of each PDX piece is not standardised, and the precise cell composition of each specific piece is unknown. Additionally, because the PCa cells are serially propagated in animals as tissue pieces, new molecular technologies that facilitate genetic manipulation, such as those that enable cell tracking or barcoding^9^ are difficult to exploit. Such manipulations would be enhanced if the PDX cells could be digested into a single cell suspension and briefly manipulated in vitro*.*

One of the reasons that PDX are not digested into a singel cell suspension, and manipulated in vitro, is that this action can compromise the viability of the PDX cell population. Recently, 3D organoid culture has been shown to enhance the survival and propagation of primary PCa cells in vitro^10,^ and that 3D culture can enhance the viability of PCa cells derived from established PDX when these cells are cultured in vitro ^11^. Both of the referenced methods allowed PCa cells to aggregate spontaneously on non-adherent tissue culture plastic^10,11^, yielding heterogeneously sized organoids. These methods identified 3D cell aggregation as a key mediator of PCa cell survival in vitro, while the resulting heterogeneity of organoid size in these culture systems identifies an opportunity for technological improvements. Microwell platforms are one of the most efficient tools avaliable to generate uniformly sized organoids, and have a proven track record of being used to generate uniformly sized organoids from embryonic stem cells, mesenchymal stromal cells, and various cancer cell lines^12–17^. The literature frequently uses the terminology organiod, spheriod or microtissue interchangeably. Because of the cell types and manipulations used herein, we favour using the terminology "microtissue". In most microwell platforms the microwell patterned surface enables multiple replica microtissues to be assemble. A weakness with these platforms is that microtissues can be displaced from microwells during media exchange, resulting in loss of microtissues or amalgamation of adjacent microtissues, and thus loss of the desired microtissue homogeneity. We previously described a microwell platform (the Microwell-mesh^18,19^), in which a nylon mesh bonded over the microwell openings retained microtissues within discrete microwells during complex medium exchanges or culture manipulations. The openings (36 µm) in the nylon mesh were large enough to allow single cell suspensions to be centrifuged through the mesh and pelleted in the bottom of the microwells during innitation of culture. However, the resulting self-assembled microtissues were too large to pass back through the mesh and remained trapped in discrete microwells over the culture period, and during complex culture manipulations.

Herein, we optimised the Microwell-mesh platform to assemble uniformly sized microtissues in vitro, and then implanted the Microwell-mesh with microtissues into NOD-scid IL2γ^−/−^ (NSG) mice to study their behaviour in vivo*.* NSG mice were used, as these animals have replaced the NOD-*scid* mouse as the most commonly used immune-compromised mouse model for xenograft studies^20^. First, we assessed vascular penetration through the mesh in vivo using Microwell-mesh platforms containing microtissues formed from bone marrow-derived stromal cells (BMSC, hereafter referred to as microtissues). Well characterised BMSC were used in this step to because these cells were more accessible than PDX-derived cells. Microwell-mesh with mesh having either 36 µm or 100 µm square openings were compared. The 100 µm mesh was more efficiently penetrated by blood vessels when implanted in the NSG mice, and subsequent in vivo experiments were all performed using this mesh size. PCa cells were enriched from three different established PDX lines, LuCaP141^21^, LuCaP35^22^ and BM18^23^, representing primary prostate cancer, lymph node metastasis, and bone metastasis, respectively. Microtissues were formed from enriched PCa cell populations (hereafter referred to as microtumours). Microtumour growth in vitro and in vivo was characterised. Proof-of-principle studies were performed to assess strategies to monitor microtumour growth using live animal imaging techniques, including ultrasound and bioluminescence imaging.

## Materials and methods

### Microwell fabrication

**Figure 1:**
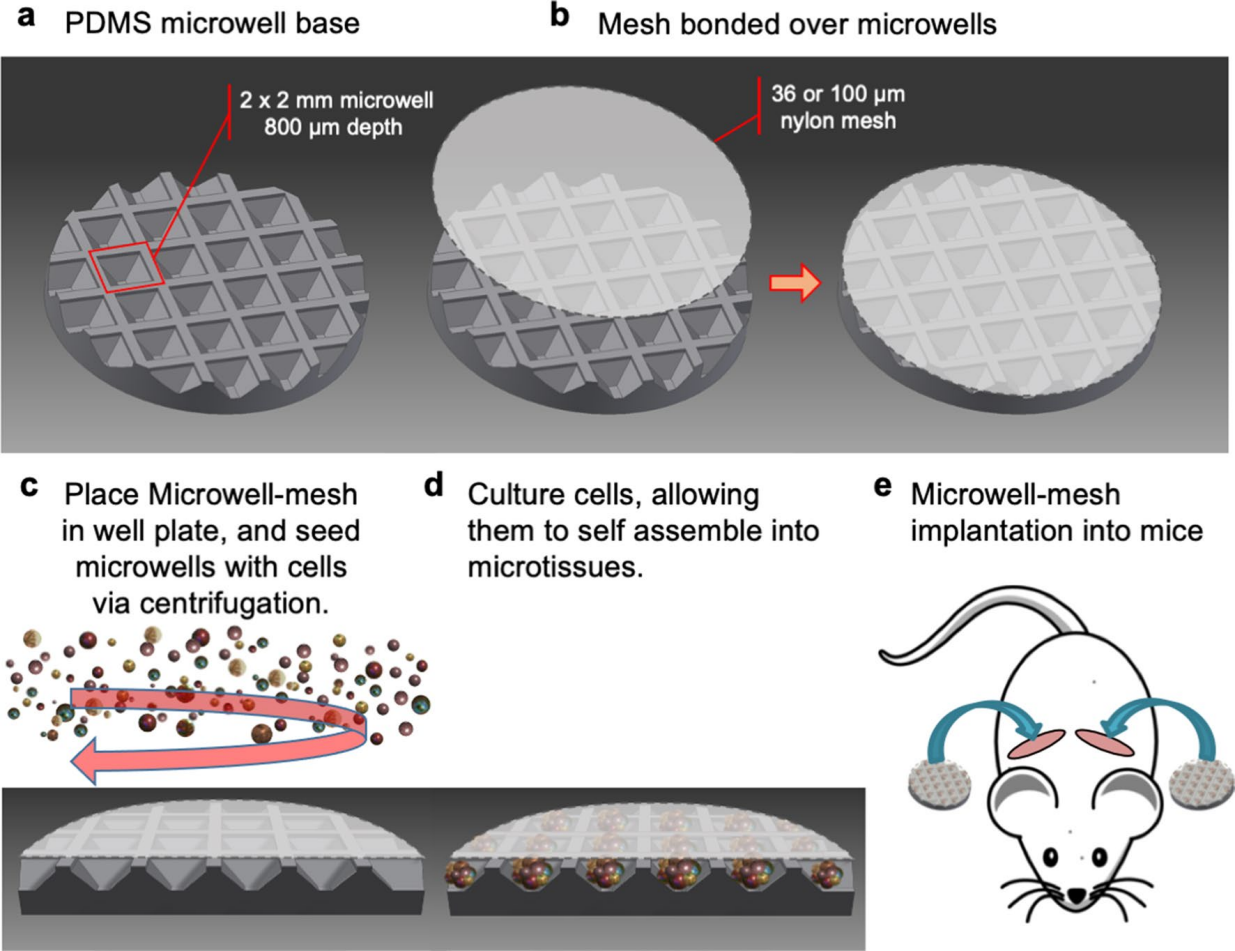
Microwell-mesh platform assembly, cell seeding, and implantation in mice. (**a**,**b**) The Microwell-mesh consisted of an array of microwells fabricated from PDMS with a nylon mesh bonded over the microwell openings. Fabrication of the Microwell-mesh is described in detail here^18^, and an animation demonstrating its capacity to retain cell spheroids is provided here^24,25^. (**c**,**d**) A single cell suspension was pelleted into the microwells by centrifugation, forming multiple microtissues, or microtumours at the bottom of the microwells. (**e**) The entire microwell-mesh platform, containing microtissues was transplanted subcutaneously onto the back of immunocompromised NSG mice. Images c-d were drawn in AUTOCAD, and image e was adapted from a free pixabay vector drawing^28^.

Fabrication of the Microwell-mesh is described in detail here^18^, and an animation demonstrating its capacity to retain cell spheroids was previously published here^24,25^. Microwells were fabricated by casting sheets of polydimethylsiloxane (PDMS; Dow Corning Sylgard 184) on a polystyrene mould^18,26,27^. Microwell features were either 360 μm × 360 μm wide × 180 μm deep (small), 800 µm × 800 µm wide × 500 µm deep (medium), or 2 mm × 2 mm wide × 800 µm deep (large). One-centimetre diameter discs were punched from the PDMS sheet. For Microwell-mesh platforms implanted in mice, large microwells were used, yielding a disc with 16-to-20 intact microwells on a each 1 cm diameter disc (Fig. 1). Nylon mesh (6/6, Amazon.com), with either 36 µm or 100 µm square openings, was bonded over microwell openings using silicone glue (Aquarium Safe, Selleys). Microwells (without mesh) or Microwell-mesh inserts were anchored into 48-well tissue culture plates (Corning) with a small dab of silicone glue. Plates were sterilised using 80% (v/v) ethanol. Bubbles were displaced from microwells during sterilisation by spinning the plates with solution at 2,000 *xg*. Plates were dried overnight at 60 °C and stored at room temperature. To prevent cell attachment to PDMS^15,27^, microwells were soaked with sterile 5% Pluronic F-127 (Sigma-Aldrich) solution for 10 min and rinsed 3 times with sterile PBS.

### Microwell-mesh optimisation of mesh size for in vivo studies

Relative vascularisation of BMSC microtissues was characterised for Microwell-mesh platforms having mesh with either 36 µm or 100 µm square openings (Fig. 1b). Expanded BMSC were used for these studies as these cells were readily available in large quantity, compared to less accessible PDX-derived cells. BMSC were isolated and cultured as described previously^18,27^. Bone marrow aspirates were collected from volunteer donors who provided informed written consent. Based on National Health and Medical Research Council (NHMRC) guidelines, ethics approval was granted by the Mater Hospital Human Research Ethics Committee (Ethics No. 1541A) and the QUT Human Research Ethics Committee (Ethics No. 1000000938). Aspirates were from a 27-year-old male (Donor 1) and a 23-year-old male (Donor 2). BMSC were cultured in low glucose (LG)-DMEM (ThermoFisher Scientific) supplemented with 1X GlutaMAX, 10% FBS, 100 U/mL PenStrep (all from ThermoFisher Scientific) and 10 ng/mL fibroblast growth factor-1 (FGF-1; Peprotech). Cells were expanded in a hypoxic (2% O_2_, 5% CO_2_) incubator at 37 °C. Medium was exchanged every 3–4 days until monolayers reached approximately 80% confluence. Cells were passaged using 0.25% trypsin/EDTA (ThermoFisher Scientific) and sub-cultured at 2 × 10^5^ BMSC per T175 flasks.

BMSC were characterised for standard mesenchymal and haematopoietic makers using flow cytometry. In brief, cells were labelled with fluorescent-conjugated antibodies or isotype controls for CD44 (Cat #130-095-180), CD90 (Cat #130-095-403), CD105 (Cat #130-094-941), CD73 (Cat #130-095-183), CD146 (Cat #130-092-849), CD45 (Cat #130-104-517), CD34 (Cat #130-090-954) and HLA-DR (Cat #130-095-298) (all Miltenyi Biotec) as per the manufacturer’s instructions. Following staining, 2 × 10^5^ cells were resuspended in 200 µL of MACS buffer (Miltenyi Biotec) and analysed on a BD LSRII flow cytometer (Becton Dickinson (BD)). FlowJo software (BD) was used to analyse data.

BMSC osteogenic and adipogenic differentiation capacity was validated using traditional 2D cultures, and chondrogenic capacity was validated using 3D pellet cultures, using methods and medium formulations as previously described^27,29^. For osteogenic and adipogenic induction, monolayers were established from 10,000 cells/cm^2^ seeded into 48-well plates, while chondrogenic pellets were formed from 2 × 10^5^ BMSC in 15 mL conical falcon tubes. Induction medium was exchanged every two days over a 21-day culture period. Cultures were fixed in 4% paraformaldehyde (PFA; Sigma-Aldrich) and assessed for differentiation. Osteogenic cultures were stained with Alizarin Red S for mineral deposition, adipogenic cultures were stained with Oil Red O for the presence of lipid vacuoles, and chondrogenic pellet tissues were embedded in O.C.T. (Tissue-Tek), sectioned (7 µm), and stained with Alcian blue for glycosaminoglycans (GAG).

To assemble BMSC microtissues in the Microwell-mesh, each well was seeded with 2.5 × 10^5^ BMSC or ~ 10,000 cells per microtissue. BMSC were suspended in high glucose (HG)-DMEM, supplemented with 10% FBS and 100 U/mL PenStrep, and pelleted into microwells by centrifuging the Microwell-mesh plate for 3 min at 500 *xg* (Fig. 1c,d). BMSC were cultured for 24 h to allow the cells to self-assemble into microtissues, and then Microwell-mesh were implanted into NSG mice (Fig. 1e), permitted to incubate in vivo for 4–8 weeks, and then harvested from mice to evaluate relative vascularisation. The diameter of vessels that formed networks through the mesh and into the microwells was quantified using ImageJ (National Institutes of Health). In all subsequent experiments, mesh with 100 µm square openings was used.

### Microwell-mesh implantation into mice

All animal procedures were approved by the UQ and the QUT Animal Ethics Committees (UQ Ethics # 441/15, QUT Ethics 1500000051), which adhere to the National Health and Medical Research Council of Australia guidelines. NSG mice were purchased from the Jackson Laboratory and bred at the Translational Research Institute in Brisbane (breeding ethics approval AEMAR62825). Male mice were used because of the intent to study prostate cancer. No animals were excluded from the studies. These studies were not blinded (i.e. it was known which cell type was implanted into which animal). Mice selected for implantation were 4–6-week-old male mice as available from breeders. Cages were maintained in a similar location to avoid confounding factors, changed by animal house staff every 2 weeks with the same diet and water. All mice were euthanised at 8 weeks, unless tumours grew beyond limit predetermined by the ethics committee. Mice were anesthetised with a 15 to 30 µL solution containing 100 mg/mL ketamine and 20 mg/mL xylazine solution delivered via intraperitoneal (IP) injection. The backs of the mice were shaved, sterilised with iodine, and two separate 2 cm × 1.5 cm subcutaneous pouches were created on the animals’ upper back. Microwell-mesh inserts containing microtissues were implanted with the mesh openings orientated ventrally. Wounds were closed using 7 mm staples and VetTech Bond.

### PDX digestion and mouse cell depletion

All original PCa PDXs (LuCaP35^22^, LuCaP141^21,30^, and BM18^23^) used in these studies were maintained using the pieces propagation method in male severe combined immune-deficient (SCID) mice. Mice bearing PDX tumours were randomly selected to collect material from to be used in this study. The confounder of cage location was not controlled. SCID mice used in PDX colony maintenance were permitted to acclimatise at the institutional animal house for a minimum of 6 days before use. Experiments were performed with the approval of the University of Queensland (UQ) and Queensland University of Technology (QUT) Animal Research Ethics Committees (Ethics # 370/17 and QUT1800000289). To generate single cell suspensions, PDX tissue were minced and processed in a GentleMACS (Miltenyi Biotec) with a maximum of 750 mg of tissue per 10 mL of digestion buffer. Digestion buffer contained 100 U/mL type IV collagenase (Worthington), 25 µg/mL DNASE II (Sigma-Aldrich), 1% Primocin (ThermoFisher Scientific), and 2.5 U/mL hyaluronidase (Sigma-Aldrich) suspended in LG-DMEM (ThermoFisher Scientific). Tubes were placed in an incubated orbital shaker at 37 °C and 200–220 RPM for 15 min, and then into the GentleMACS for 60 s at a mid-range RPM. If solid tumour parts were visible, tissues were treated for an additional 10 min at 37 °C with gentle rocking, followed by GentleMACS for 60 s on a spleen dissociation cycle, followed by 30 s at high RPM. Cell digests were suspended in a chilled solution of 1:1 LG-DMEM and FBS, filtered through a 40 µm strainer, centrifuged at 550 *xg* for 5 min at 4 °C, and washed with chilled PBS + 10% FBS. Cells were resuspended in 5 mL of red blood cell lysis buffer (ThermoFisher Scientific), and gently agitated for 7 min at room temperature, centrifuged at 550 *xg* for 5 min at 4 °C, and resuspended in chilled PBS + 2% FBS. Single cells were counted using an automated cell counter (Biorad TC20) gated at ≥ 6 µm in diameter. The Mouse Cell Depletion Kit (Miltenyi Biotec, Cat No. 130-104-694) was used to enrich the digest for PCa human cells by depleting murine cell populations including fibroblasts, immune cells, and endothelial cells from the PDX digests. Previous studies have used this kit for similar applications^31,32^. Approximately 5 × 10^7^ dissociated PDX cells were resuspended with 80 µL of cold separation buffer (0.5% BSA, 100 U/mL PenStrep in PBS) and 20 µL of Mouse Cell Depletion cocktail, and then incubated for 15 min at 4 °C, with occasional agitation. The total volume was adjusted to 500 µL per 10^7^ cells, and the suspension was run through the autoMACS Pro Separator (Miltenyi Biotec). Both tumour cell and mouse cell populations were collected and washed twice in cold 2% FBS in PBS.

### Flow characterisation of enriched PDX PCa cell populations

PDX PCa cells were stained with antibodies reactive against human PSMA (hPSMA; APC, Biolegend, Cat #34250, Clone LNI-17), human CD44 (hCD44, Miltenyi Biotec, Cat #130-095-180), human EpCAM (CD326) (APC; Biolegend, Cat #324208, Clone 9C4), and mouse CD45 ((mCD45; APC-Cy7, BD Becton Dickinson, Cat #557659, Clone 30-F11), as well as a viability discriminator dye, 7-AAD or Live Dead Aqua (ThermoFisher Scientific) as per the manufacturers’ instructions. Single colour controls were prepared for human and mouse reactive antibodies from the mouse cell-depleted and mouse cell-replete PDX-derived cell fractions, respectively. Mouse CD45 beads (Miltenyi Biotec) were used for positive controls. Negative controls were generated by staining the respective cell fractions with isotype controls. Flow cytometry was performed on a LSRII flow cytometer, and data analysed with FlowJo software.

### In vitro culture of PDX-derived PCa cells in 2D and 3D

PDX-derived PCa cells were cultured in medium previously developed by Hans Clevers’ laboratory for prostate cancer organoid cell culture^10,33^, hereafter referred to as Hans Clevers’ Medium (HCM). Medium components are listed in Supplementary Table 1. Each Microwell-mesh platform was seeded with 2.5 × 10^5^ cells in 1 mL of HCM, yielding approximately 10,000 cells per microwell, or 10,000 cells per microtumour. In cases where in vitro observation was the primary objective, rather than implantation into animals, microtumours were cultured in microwell platforms without mesh. Parallel 2D monolayer cultures were seeded at the same cell density (2.5 × 10^5^ cells/cm^2^) in standard 48 well plates to compare the viability of PDX cells in 2D and 3D. Cells were incubated at 37 °C, 20% O_2_, and 5% CO_2_, and the viability of both 2D and 3D cultured PDX cells assessed using Live/Dead stain (Thermo Fischer Scientific) as per the manufacturer’s instructions.

### In vivo studies with PDX PCa cell microtumours in the Microwell-mesh

Microwell-mesh and microtumours were prepared as above, and incubated overnight at 37 °C, 20% O_2_, and 5% CO_2_ to allow the cells to self-assemble into microtumours before being implanted into mice, as described for BMSC microtissues.

### In vivo Matrigel control studies

As a control, dissociated PDX-derived PCa cells were implanted into mice using Matrigel as a carrier. Cells were cultured overnight in HCM, and then ~ 2.5 × 10^5^ cells were suspended in 50 µL of chilled HCM plus 50 µL of Matrigel. Mice were prepared as described above, and then the suspension of cells in Matrigel injected subcutaneously into the lower flanks of NSG mice.

### In vivo imaging using the VEVO LAZR

Ultrasonic visualisation uses high frequency 3D ultrasound and photoacoustic images to characterise tissues and blood flow. Data was acquired using Vevo 2100 (VEVO LAZR, FUJIFILM, VisualSonics) at 4 and 8 weeks following Microwell-mesh/microtumour implantation. Mice were anaesthetised as described above, side flanks and backs shaved, and remaining fur removed using hair removal cream (Nair). Anaesthesia was maintained during imaging using 2% O_2_ ventilated isoflurane. Areas of interest were placed directly below the transducer with a layer of Clear Ultrasound gel (ThermoFisher Scientific). 3D volumetric visualisation was achieved by rendering B-Mode scans in Vevo LAB 3.2.6 software. Full volumes were acquired from end to end of tumours by identifying the tumour edge manually, and setting full scan with a step size of approximately 0.2 mm. Averaging was used to gate out animal respiration artefacts.

### Transduction of dissociated PDX-derived cells and IVIS imaging

PDX-derived PCa cells were transduced to express luciferase driven by an EF1-alpha promotor, using our previously described protocol^34^. In brief, plasmids were manufactured in Stbl3 Competent *E. coli* (Invitrogen), purified using a NucleoBond Xtra EF plasmid purification kit (Midi EF, Macherey–Nagel), and transfected into HEK293FT cells (Invitrogen) using Lipofectamine 2000 (Invitrogen). Viral particles were collected from HEK293FT conditioned medium, and used to transduce PDX-derived PCa cells. Plates (6 well, Nunc) were coated with fibronectin (BD Biosciences, 10 µg/mL for 1 h) and then inoculated with 2 × 10^6^ PDX-derived cells in 0.5 mL HCM and 0.5 mL viral particle conditioned HCM plus 6.5 µg of polybrene (Sigma-Aldrich). After 24 h of exposure to viral particles, the medium was exchanged with fresh HCM, and the culture continued for 24 h. Luciferase activity was assessed in transduced cells by supplementing cultures with 150 µg/mL D-Luciferin (Elmer XenoLight, Perkin Elmer), incubating for 2 min, and measuring the bioluminescent signal using the Spectrum In Vivo Imaging System (IVIS, PerkinElmer).

Mice were imaged directly following Microwell-mesh or Matrigel implantation, and then approximately every 7 days to monitor growth. Prior to imaging, mice received an IP injection of 100 µL D-Luciferin in PBS (150 mg D-Luciferin per kg of mouse body weight). During acquisition, isoflurane anaesthetised mice were laid on their sides between dividers and bioluminescence scanned. Regions of interest (ROIs) were drawn on the first captured scan around the areas containing PDX cells to monitor bioluminescent signal. Images were then continuously captured on auto-exposure at the standard animal height setting every 2 min until maximum peak had been reached in the ROIs. Peak time was estimated by identifying the time after which there was two to three consecutive images of decreased overall radiance/flux captured in the drawn ROIs. Using ROIs, the total flux in photons/second was estimated for each animal.

### Harvesting tumours from animals

Microwell-mesh containing microtumours were harvested at 4 weeks during optimisation, with subsequent harvests performed at 8 weeks. If the tumour mass exceeded 2 cm^3^, harvest was performed earlier. Animals were euthanised by CO_2_ inhalation and cervical dislocation. PDX-derived microtumours or Matrigel controls were harvested.

### Histological processing and staining

Tissues were fixed in 4% PFA for 24 h, dehydrated with an ascending ethanol series followed by xylene, and paraffin embedded. Tissues were sectioned at 4–6 µm and collected onto SuperFrost slides (ThermoFisher Scientific). Sections were stained with haematoxylin and eosin (H&E), and with anti-PSA (Ventana Cat #: 760-2506) and anti-Ku70 (ab58150) antibodies. For antibody staining, sections were treated with peroxidase blocking reagent (Dako, S2001) for 5 min at room temperature, antigen-retrieved (10 mM sodium citrate, pH 6.0) at high temperature/pressure for 1 min, and blocked with Protein Block Serum Free Medium (Dako, X0909) for 30 min at room temperature. Primary antibodies (anti-PSA at 1:100, and anti-Ku70 at 1:6000) in 0.5% BSA were incubated on the slides overnight at 4 °C. The DAKO LSAB2 detection system (Cat #K0675) was used per manufacturer’s instruction to secondarily label the primaries. A DAB substrate kit (abcam, K3466) was used to develop the chromogenic signal. Sections were counterstained with Mayer’s hematoxylin, followed by cover-slipping in DePex mounting media (Electron Microscopy Sciences, Fort Washington, PA, USA). Negative controls were prepared by substituting primary antibody with mouse IgG1 (Dako, X0931).

### Ethics

Animal procedures were approved by the UQ and the QUT Animal Ethics Committees, which adhere to the National Health and Medical Research Council of Australia guidelines, and the study is in compliance with the ARRIVE guidelines where animals are involved.

## Results

### BMSC characterisation and selection of mesh opening size

Supplementary Figs. 1a–c and 2a–c show successful tri-linage differentiation of BMSC donors 1 and 2. BMSC formed adipocyte-like cells that contained lipid vacuoles (stained with Oil Red O), osteoblast-like cells that mineralised (stained with Alizarin red S), and chondrocyte-like cells that generated GAG-rich matrix (stained with Alcian Blue). Supplemental Figs. 1d and 2d show that BMSC donors 1 and 2 stained positively for mesenchymal markers CD44, CD73, CD90, and CD105 and negative for hematopoietic cell markers CD34, CD45, and HLA DR^27,29^.

**Figure 2:**
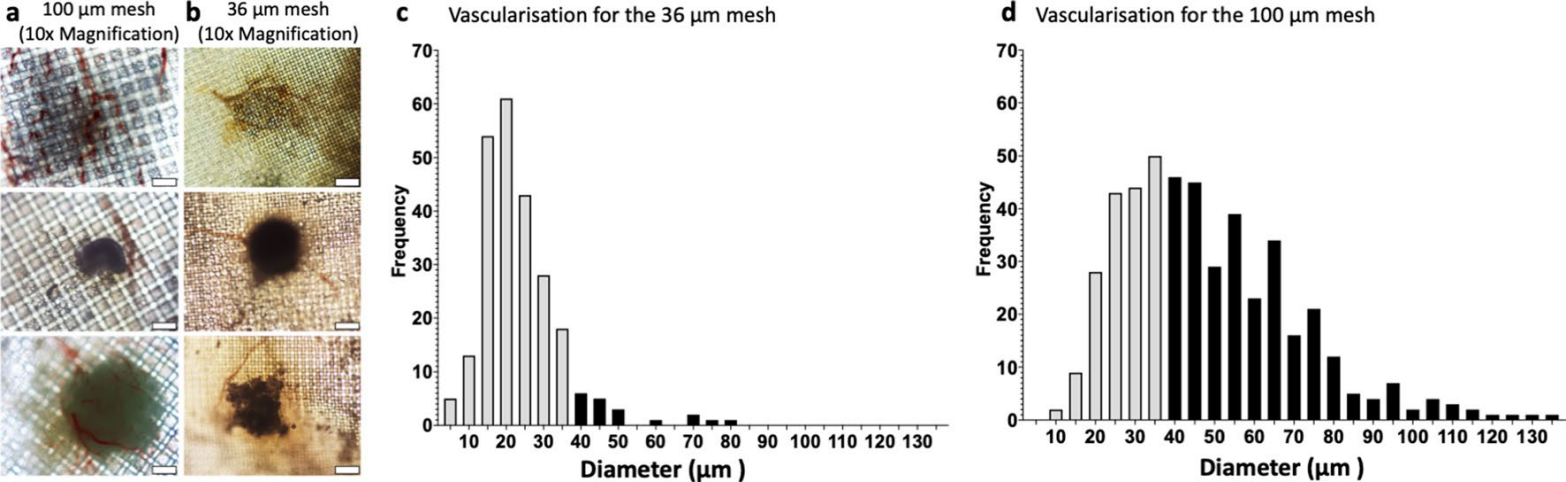
Comparison of vascularisation through a 36 µm or 100 µm mesh. Representative images of vascularisation of BMSC microtissues through the (**a**) 100 µm mesh and through the (**b**) 36 µm mesh (scale bars = 200 µm). Vascular network diameter characteristics for vessels observed feeding microtissues in Microwell-mesh with (**c**) 36 µm mesh size openings (n = 35) and (**d**) 100 µm mesh size openings (n = 35). Grey bars represent blood vessels < 35 µm in diameter and black bars represent blood vessels > 35 µm. Smaller mesh openings would have excluded penetration of the many vessels having diameters greater than > 35 µm, while larger mesh sizes would have theoretically enabled vessels up to 100 µm to pass through. Data was collected from images of Microwell-mesh inserts that had been implanted into animals, harvested, imaged, and then analysed manually using ImageJ.

BMSC donors 1 and 2 were assembled into microtissues using Microwell-mesh having mesh with either 36 µm or 100 µm square openings and implanted in mice (Fig. 1) for 4 or 8 weeks. Microscopy images (Fig. 2a,b) were used to characterise the vasculature feeding through the mesh and into the microwells, and vessel diameters are displayed graphically in Fig. 2c,d. The vascular structures penetrating the 36 µm meshes appeared less extensive than those penetrating the 100 µm mesh openings. More large-diameter vessels were observed when the 100 µm mesh was used compared with the 36 µm mesh (Fig. 2c,d). Many of the vessels observed on the surface of the 100 µm mesh would not have easily penetrated the 36 µm, without reducing their diameter or restricting at the point of penetration. Based on these data the 100 µm nylon mesh was used in the fabrication of Microwell-mesh for subsequent studies.

### Characterising PCa cells from PDX digests

Each PDX type (LuCaP35, LuCaP141, and BM18) had a characteristic flow cytometry forward scatter and side scatter profile, as shown in Supplementary Fig. 3a–c, respectively. Of the three PDX lines used, LuCaP35 isolations yielded the largest cells, identifiable by a higher forward scatter on the flow cytometer and visibly larger when observed using a microscope. LuCaP141 and BM18 forward scatter was lower, and these cells could not be discriminated from mouse cells without the use of antibody markers. Before and after mouse cell depletion, the fraction of mCD45 was quantified, and hCD44, EPCAM and hPSMA expression was characterised on mouse cell depleted populations. These results are presented in Table 1, and the flow cytometry data shown graphically in Supplementary Fig. 3. These data indicate that while the marker profile was variable on different PDX populations, mouse cell depletion successfully enriched for human PCa cells.

**Table 1:** Marker expression before and after mouse cell depletion.

PDX	mCD45 prior to mouse cell depletion (%)	Post mouse cell depletion			
		mCD45 (%)	hCD44 (%)	EPCAM (%)	hPSMA (%)
LuCaP35	73.8 ± 19.2	13.0 ± 7.1	77.7 ± 9.7	90.8 ± 7.6	33.1 ± 17.2
LuCaP141	68.5 ± 6.3	15.4 ± 8.4	46.2 ± 22.1	78.9 ± 11.2	62.8 ± 23.7
BM18	76.4 ± 16.9	26.0 ± 5.5	53.4 ± 7.9	93.0 ± 3.0	75.1 ± 20.7

Values represent averages ± standard deviations. LuCaP35 analysis from n = 6 PDX tumours, LuCaP141 analysis from n = 5 PDX tumours, and BM18 analysis from n = 3 tumours. Graphical data is provided in Supplementary Fig. 3.

### Monolayer culture of PDX PCa cells

**Figure 3:**
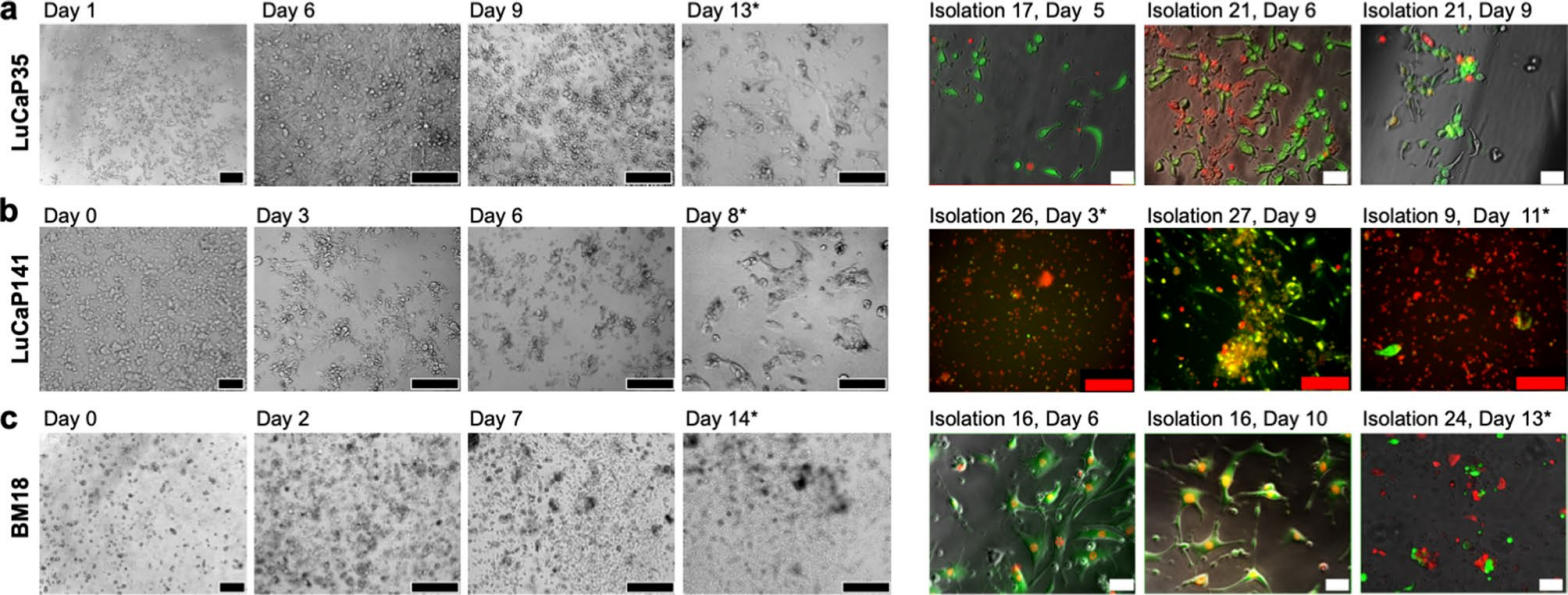
Mouse cell-depleted PDX PCA cells in monolayer culture in HCM. (**a**) LuCaP35, (**b**) LuCaP141, and (**c**) BM18. Representative viability staining is shown from different PCa cell isolations and timepoints. Live cells are green (Calcein AM), and dead cells are red (Ethidium Homodimer-1). Black and white scale bars = 50 µm, red scale bars = 200 µm. Asterisks indicate the timepoint where cultures were terminated and stained because of poor viability.

PCa cells isolated from LuCaP35, LuCaP141 and BM18 PDX, and depleted of mouse cells, were cultured in 2D monolayer in HCM for up to 14 days. Bright field images were captured, as well as fluorescent images of cells stained with viability dyes (Fig. 3). Over time, cells in monolayer culture shrunk in size, declined in numbers, and viability dyes indicated an increase in the number of dead cells (red) relative to the number of live cells (green). Supplementary Fig. 4 provides viability data from representative cultures characterised over time. While a small subset of BM18 and LuCaP35 PCa cells remained viable for greater than 14 days, overall cell number declined with time. These data indicate that these PDX-derived PCa cells do not proliferate in these 2D culture conditions, which parallels the reported inability to expand primary PCa cells in monolayer culture^10^.

### Microtumour culture of PDX PCa cells

**Figure 4:**
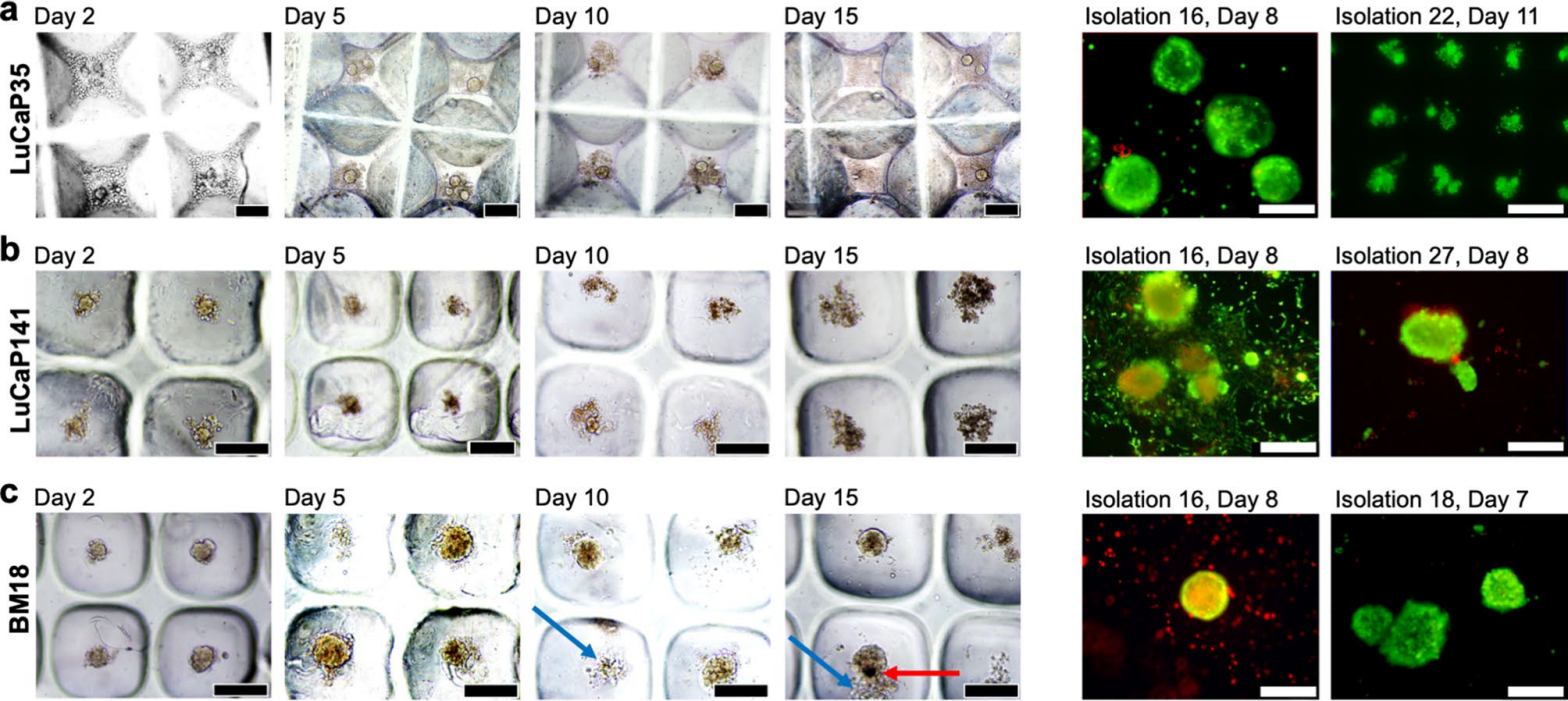
Mouse cell-depleted PDX PCA cells in 3D microtumour culture. (**a**) LuCaP35-derived cells formed small aggregates, sometimes requiring more than a single day to amalgamate into a single microtumour per well. (**b**) LuCaP141-derived cells were the least consistent in forming a single discrete microtumour per microwell. Frequently small clumps or loose cells would surround each microtumour. (**c**) BM18-derived cells formed the largest microtumours. While none of microtumours consistently increased in size during in vitro culture, cells did remain viable (dye Live(green)/Dead(red)) over several days. Red arrow points to a region of a microtumour that has become tightly aggregated and compacted, while the blue arrows point to cell debris that has not been incorporated into the spheroid. Scale bars = 200 µm.

PCa cells assembled into 3D microtumours in microwells remained viable for a greater period of time than 2D monolayers, but cell number did not appear to increase (Fig. 4a–c). Supplementary Fig. 5 shows microtumour diameters prior to implantation. LuCaP35 microtumours were 364 ± 42 µm in diameter (n = 75), while LuCaP141 microtumours were 325 ± 63 µm in diameter (n = 52), and BM18 microtumours were 242 ± 68 µm in diameter (n = 46). The average diameter of microtumours was greater than 100 µm and thus they were retained within discrete microwells by the 100 µm mesh. In some cases, LuCaP141 PDX cells did not form a single continuous spheroids in each microwell, and instead formed multiple small spheroids with some loose cells piled proximally (Fig. 4b). In addition to the viability staining, the maintenance of intact 3D microtumours was indicative of greater cell survival in 3D microtumours relative to 2D cultures. Over extended culture time, cell viability declined, and spheroid compaction was observed (red arrow). Dark cores formed in compact microtumours while cell piles or clumps became looser, composed of shrinking cells and accumulating cell debris (blue arrows). While superior to 2D monolayers, 3D spheroid culture in HCM was insufficient to support proliferation of these PDX-derived PCa in vitro. However, the improved short-term in vitro viability of PDX-derived PCa cells in 3D micro-tumours indicated that PDX could be digested into a single cell suspension, depleted of mouse cells, and viability maintained in short-term cultures (7–14 days) if the cells were in the described 3D organisation.

### Microtumour in vivo establishment rates and characteristics

**Figure 5:**
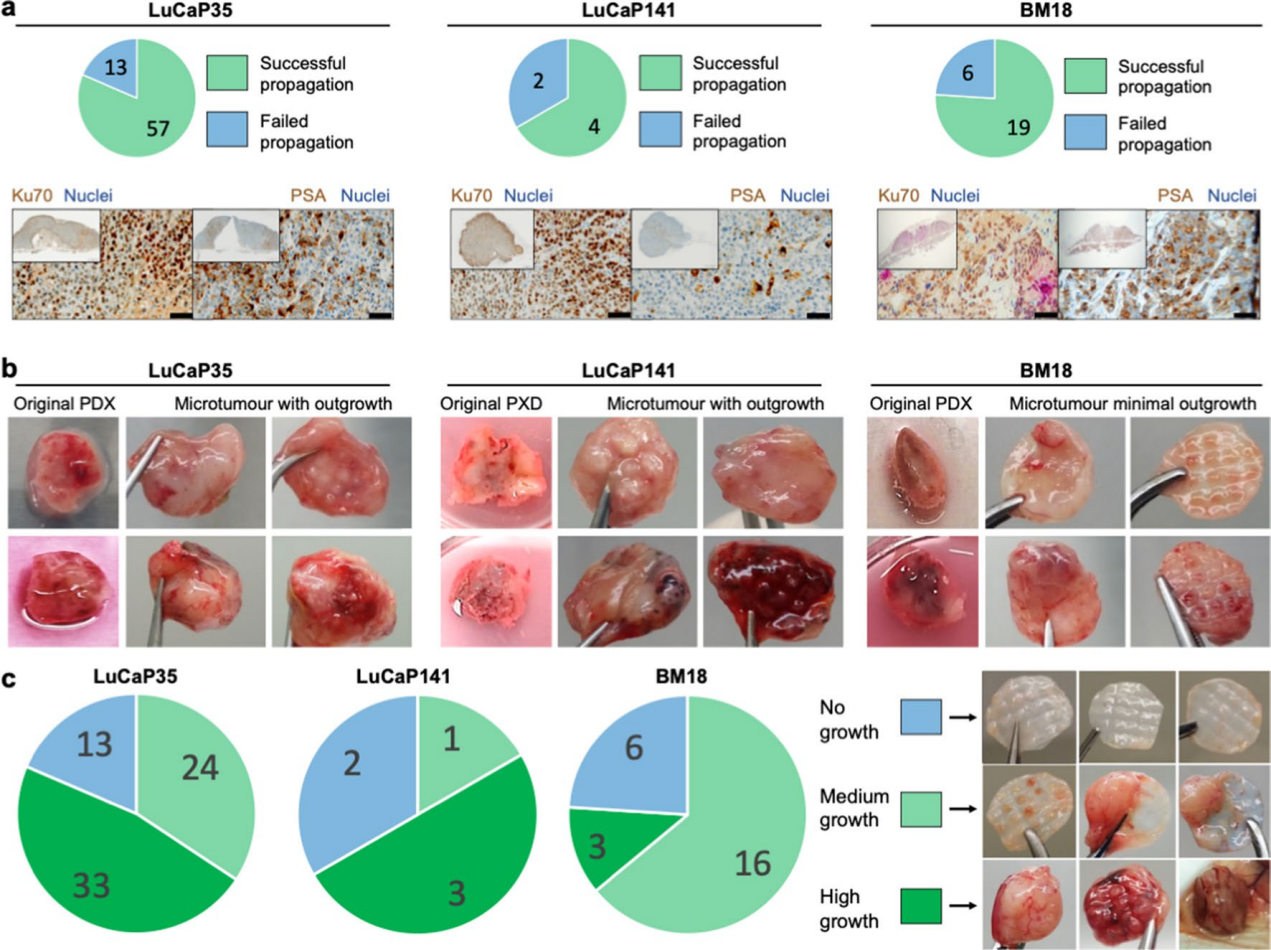
Microtumour establishment rates and histological comparison to original PDX. (**a**) Rates of microtumour establishment with different PDX-derived PCa cell populations using the Microwell-mesh platform. Immunohistochemistry staining of tissues derived from microtumours for human cell-specific Ku70 and PSA. Scale bars = 50 µm. (**b**) Representative images of parental PDX tumours and the tissues harvested following PDX re-establishment in mice using the Microwell-mesh platform, after 8 weeks in vivo. (**c**) The number of tumours recovered from transplanted microtumours showing “No growth”, “Medium growth”, and “High growth” are shown in pie charts for each PDX population, as well as 3 representative photographic images.

In our hands, the parental PDX have been passaged successfully for several years using pieces with 97.5%, 92.1%, and 97.2% take rates for LuCaP35, LuCaP141, and BM18, respectively. The success rate for re-establishing PDX-derived cells as *micro*-tumours in mice using the Microwell-mesh platform was as follows (Fig. 5): 57/70 (81%) for LuCaP35, 4/6 (67%) for LuCaP141, and 19/25 (76%) for BM18 (Fig. 5a). Figure 5b provides examples of histological sections of microtumours that outgrew the mesh to demonstrate that the enriched cell populations yield common tumour morphology that is populated by human cells (Ku70^+^). Representative H&E staining for each microtumour PDX population is provided in Supplementary Fig. 6. Tumor establishment in mice from single cell suspensions in Matrigel was 2/4 (50%) for LuCaP35 and 1/5 (20%) for BM18. This low success rate in Matrigel is similar to historical experience in our laboratory; because of this low success rate, subsequent animal resources were focused on efforts to characterise microtumour propagation in the Microwell-mesh. The different microtumour behavior at 8 weeks is further stratified in Fig. 5c. LuCaP35 had the highest establishment rate, and by 8 weeks many microtumours (33/70, 47%) had outgrown the Microwell-mesh, forming a large tumour mass. At 8 weeks, 3/6 (50%) of LuCaP141 had outgrown the Microwell-mesh. The reduced outgrowth and reduced microtumour establishment rate with LuCaP141 may be linked to their reduced propensity to form continuous cell aggregates in vitro (Fig. 4b). At 8 weeks, BM18 microtumours were the most consistent (16/25, 64%) in the formation of viable microtumours that frequently did not outgrow the Microwell-mesh. The outgrowth rates provide an indication of the likely functional life of the microtumour model. Drug or experimental testing with the microtumour model would have to be completed before the tissues outgrew the platform and amalgamated into a continuous tissue.

### Live animal imaging of micro-tumours with the VEVO LAZR

**Figure 6:**
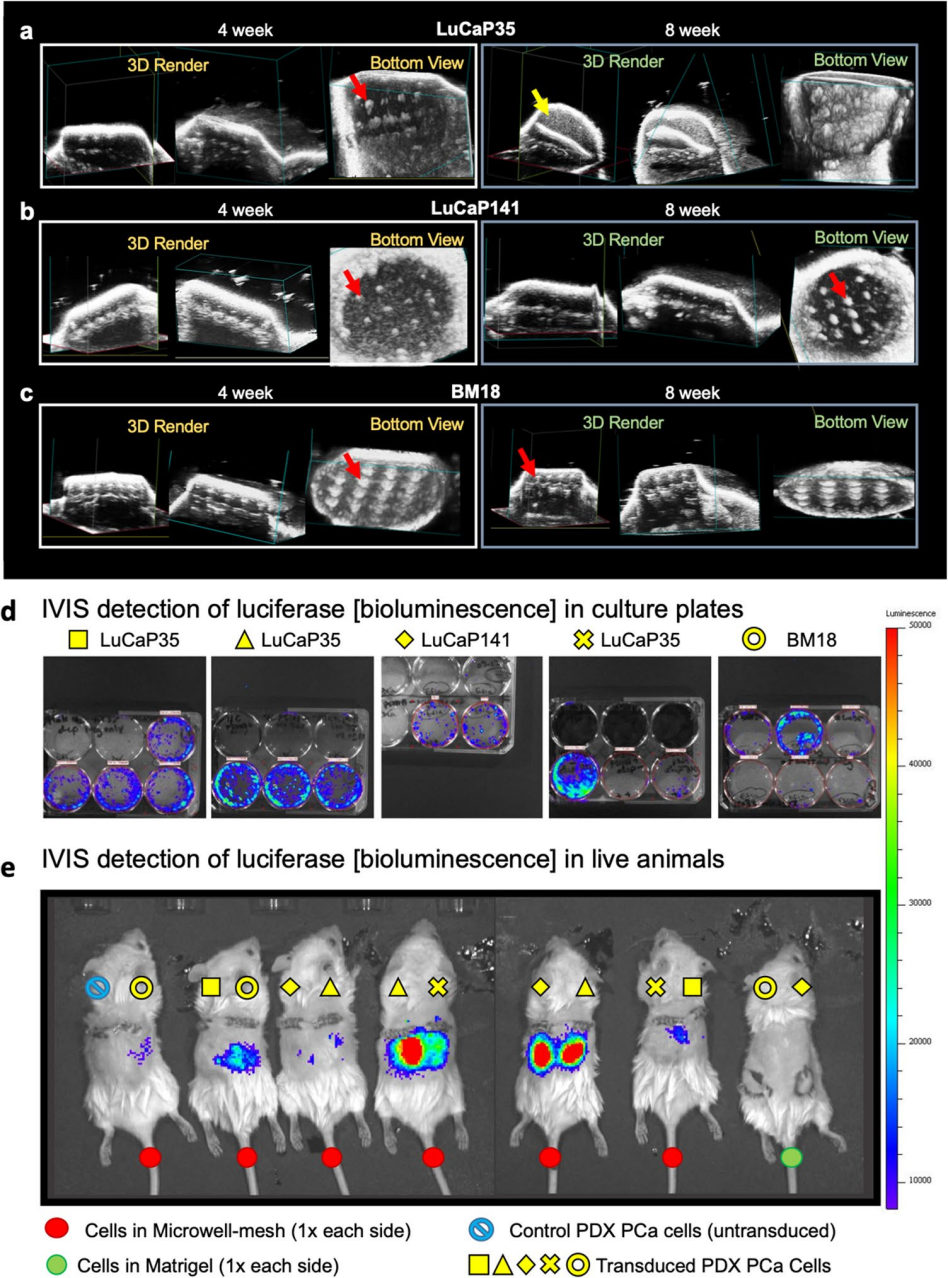
Live animal imaging of microtumours. (**a-c**) Microtumours imaged with the VEVO LAZR at 4 and 8 weeks. Microtumours either appeared as spheroidal and arrayed tissues contained within the Microwell-mesh (red arrows), or as a larger cell mass external to the Microwell-mesh platform following breaching of the Microwell-mesh (yellow arrow). (**d**) Bioluminescence signal from transduced PDX cells cultured in well plates. Each culture is assigned a yellow shape, and this aligns with the cells implanted into animals in caption E. (**e**) Bioluminescence signal from transduced microtumours in Microwell-mesh implanted into mice. Red dot corresponds to animals with Microwell-mesh implanted, green dot corresponds to an animal with transduced cells in Matrigel. Notation from caption D indicates PDX population, i.e. Yellow square = LuCaP35, Yellow triangle = LuCaP35, Yellow diamond = LuCaP141, Yellow cross = LuCaP35, and yellow circle = BM18.

VEVO LAZR ultrasound imaging was used to monitor the growth of microtumours in live mice at 4 and 8 weeks. Microtumours could be identified and clearly visualised within the Microwell-mesh by their dense spheroidal appearance and arrayed organisation (Fig. 6a–c). These data demonstrate the capacity for this imaging approach to allow tracking of microtumour growth in live animals. While this imaging method provides significant information, data capture was time consuming and tedious. Efficiency improvements will be required to make this approach feasible across groups of animals.

### Live animal imaging of microtumours with the IVIS

To visualise microtumors in live animals, cells were transduced to express luciferase. During characterisation of cells Luciferin was added to the cell culture medium, and the bioluminescent signal from the cell cultures was quantified using an IVIS (Fig. 6d). Transduced cells were seeded into the Microwell-mesh platforms, incubated in vitro overnight, and subsequently implanted into mice. When Luciferin was injected into animals, a bioluminescent signal could be detected emanating from the location where the Microwell-mesh were implanted (Fig. 6e). It was not possible to discern the signal from individual microtumours within the Microwell-mesh, likely due to light diffusion and animal movement caused by breathing. Overall, live animal imaging of luciferase expressing microtumours using the IVIS was more efficient than the VEVO LAZR, but did not provide the resolution required to characterise discrete microtumours.

## Discussion

In previous work, we described a microwell culture platform called the Microwell-mesh, and demonstrated its utility in culturing microtissues formed from BMSC or microtumours formed from cancer cell lines^18,19,35^. In previous work, the Microwell-mesh was fabricated with a mesh having 36 × 36 µm square openings, and openings of this size were suitable for in vitro culture of microtissues. Here, the aim was to implant the Microwell-mesh into mice and have mouse vasculature penetrate the mesh and nourish the microtissues contained in each microwell. Microwell-mesh fabricated with mesh having either 36 × 36 µm or 100 × 100 µm square openings were compared. BMSC microtissues cultured in the Microwell-mesh were implanted in NSG mice and vascularisation was characterised. While mouse vasculature passed through meshes of both sizes, more than half of the vessel structures that formed on the outside of the mesh were greater than 35 µm in diameter, and more developed vasculature networks penetrated the 100 µm mesh openings. Based on these observations, all further in vivo studies were completed using Microwell-mesh fabricated with the 100 µm mesh opening size.

Barriers to widespread PDX use include the challenge of sharing viable tissues, the cost of PDX propagation, and ethical considerations associated with animal use. When PDX are used, propagation is generally achieved using the pieces method, especially for PCa PDX^36,37^. While this method is robust, a method that would enable the generation of multiple PDX tumours in a single animal, each formed from precise numbers of human PCa cells would represent a significant advancement, as such a method could lead to the more cost effective and ethical use of animals. Herein, the merits of using the Microwell-mesh to assemble PDX PCa cells into uniformly sized microtumours and propagate these microtumours in vitro and in vivo was systematically evaluated.

Previous attempts to culture single cell digests of the PDX used in this study (LuCaP35, LuCaP141, and BM18) had only limited success^38^. Similarly, expansion of these cells in 2D monolayer or in 3D microtumour cultures was not observed, even in medium previously shown to support primary PCa cell growth^10^. However, when the PDX-derived PCa cells were assembled into microtumours in microwells, and cultured in HCM, cell viability was maintained for several days (7–14 days). These data suggested that assembly of disassociated tumour cells into microtumours could improve the viability of these cells in short-term culture in vitro, providing opportunity for in vitro manipulation prior to re-implantation in mice.

To determine whether the Microwell-mesh could serve as a tool to facilitate transition of dissociated PDX cells from an in vitro to an in vivo environment, we assembled microtumours in the Microwell-mesh overnight and then implanted the Microwell-mesh into mice. The success rate for microtumour formation and growth in mice was 57/70 (81%), 4/6 (67%), and 19/25 (76%), for cells derived from LuCaP35, LuCaP141, and BM18 PDX, respectively. In small parallel studies, the success rates for establishment of tumours from disassociated LuCaP35 or BM18 in Matrigel was 2/4 (50%) and 1/5 (20%), respectively. These results aligned with our historically limited success when attempting to propogate these disassociated PDX PCa cells in Matrigel. 3D microtumour culture enhanced PDX-derived PCa cell viability in vitro, similar to previously described organoid cultures^10,11^, and appeared to improve subsequent propagation of these cells in mice. Cumulatively, microtumour assembly enhanced the survival of PDX cell populations that had been digested into a single cell suspension, followed by enrichment for human cells.  Based on these outcomes, we posit that the Microwell-mesh and microtumour approach could be used to standardise the starting material used in PDX animal studies. By generating multiple replicate tissue units, each having similar cellular composition, the resolution of experiments may be increased, and the required number of animals may be decreased.

A common argument for the pieces method of PDX propagation, and a potential counter argument for the use of the microtumour model, is that the *pieces*, which include stromal cells, immune cells, and endothelial cells, retain aspects of the original tumour architecture^39^. However, it should be noted that during passaging in mice, the human support cell populations are replaced by mouse support cells. Thus, the retained PDX architecture is orchestrated by the immortal human cancer cells that are serially passaged in mice. For this reason, it may be reasonable to expect that the resulting PDX tumours, guided by the immortal PCa cells, will evolve to have a similar architecture even if initially depleted of stromal or support cell populations. While our histology data suggest this is the case, this assumption should be tested over multiple microtumour generations. Specificially, future studies should confirm that the PDX microenvironment or structure is iteratively regenerated when microtumours are used, and that drug response mirrors the parental PDX and original cancer. Both of these properties may be improved, or could be studied, by augmenting the enriched PCa cells with specific human support cells.

The ability to image the growth of discrete microtumours over time in live animals would enable the more efficient use of animals and support use of the microtumour concept. To this end, we tracked microtumour growth in live animals using ultrasound (VEVO LAZR) or bioluminescence (IVIS). VEVO LAZR generated high resolution images of discrete microtumours within the Microwell-mesh. Similarly, when PCa cells were transduced to express luciferase, these cells could be visualised in live animals with the IVIS. However, while bioluminescence signal could be detected, signal from adjacent microtumours bled into each other, preventing visualisation of discrete microtumours. Thus, while time consuming, only the VEVO LAZR provided the resolution required to track the growth of individual microtumours.

While this work establishes a basic workflow for microtumour generation and use, multiple challenges and opportunities remain for process improvement, including a more automated Microwell-mesh manufacture process, potential improvement in platform design, as well as cell labelling and imaging capabilities. The described Microwell-mesh platform was fabricated manually^19^. An automated manufacturing process would yield a more consistent product and enable distribution to laboratories that do not have fabrication facilities. The current platform’s functional utility lies over a time continuum from successful vascular integration of microtumours, to the point where they over grow the platform and amalgamate with each other (i.e. when the microtumours are no longer discrete tissues). Using the current Microwell-mesh design, this functional period is approximately 4–8 weeks. Extending the functional period may be achieved by redesigning the dimensions of the microwells (e.g. wider and/or more tapered microwells), which would minimise microtumour coalescence and permit longer studies. Integration of lab-on-chip biometric technologies or contrast agents into the Microwell-mesh platform could facilitate tracking of *micro*-tumour size or health. Efficient and quantitative imaging techniques will be necessary features of any platform that seeks to reduce animal numbers; fortunately imaging technologies are improving at a rapid pace^40^. Viral barcoding of tumour cell populations^9^ could be used to track the clonal cell growth, the loss of specific clones due to drug treatment, or the adaption of specific clones in response to a drug treatment. This paper outlines the first steps towards microtumours and Microwell-mesh platforms that have the potential to enhance the power of studies and improve the efficient use of animals.

## Conclusion

The Microwell-mesh is a unique platform that can be used to culture microtissues in vitro and directly implant the microtissue array in vivo. In optimisation studies, mouse vasculature reliably integrated with BMSC microtissues contained within the Microwell-mesh, and then further reliably vascularised and supported rapidly growing PCa microtumours. Using this refined method, PCa cells from LuCaP35, LuCaP141, and BM18 PDX were reliably propagated as arrays of microtumours in mice, and could be imaged in live animals over time. This basic workflow could facilitate more cost efficient and ethical use of animals and might be tailored to address specific biological or therapy-related questions.

The in vitro assembly and in vivo implantation of these microtissue arrays may have important tissue engineering applications beyond those demonstrated here. For example, this approach could be used to facilitate and study implantation of common spheroidal tissues such as islets^41^, hair follicles^42^, or other organ-specific cells that self-assemble into 3D structures^43^. In these applications, the Microwell-mesh could provide an important bridge between in vitro assembly of complex mini organs and in vivo implantation.

## Supplementary Information

Supplementary Information
