# Supplementary material for: Using the Microwell-mesh to culture microtissues in vitro and as a carrier to implant microtissues in vivo into mice
Source: Sci Rep. 2021 Mar 4;11:5118. doi: 10.1038/s41598-021-84154-4 (PMC7933425; doi:10.1038/s41598-021-84154-4)
Supplement: Supplementary file 1 — Supplementary Information [file 41598_2021_84154_MOESM1_ESM.docx]

**Supplementary Material**

**Using the Microwell-mesh to culture microtissues *in vitro* and as a carrier to implant microtissues *in vivo* into mice**

Melissa E Monterosso, Kathryn Futrega, William B. Lott, Ian Vela, Elizabeth D. Williams, and Michael R. Doran

**Supplementary Figures**

**Supplementary Figure 1**: BMSC Donor 1 Characterisation. (**a-c**) Validation of *in vitro* trilineage potential of BMSC Donor 1 after 21 days of induction in monolayer (osteogenic, adipogenic) and pellet culture (chondrogenic). (A) Osteogenic cultures were stained with Alizarin Red S, (**b**) adipocytes were stained with Oil Red O, and (**c**) sectioned chondrogenic pellets were stained with Alcian Blue. (**d**) Flow cytometry analysis of surface markers demonstrated that BMSC expressed CD44, CD73, CD90, and CD105 and were negative for haematopoietic cell markers CD34, CD45, and HLA DR. White scale bar = 200 μm. Black scale bar = 100 μm.

**Supplementary Figure 2**: BMSC Donor 2 Characterisation. (**a-c**) Validation of in vitro trilineage potential of BMSC Donor 1 after 21 days of induction in monolayer (osteogenic, adipogenic) and pellet culture (chondrogenic). (**a**) Osteogenic cultures were stained with Alizarin Red S, (**b**) adipocytes were stained with Oil Red O, and (**c**) sectioned chondrogenic pellets were stained with Alcian Blue. (**d**) Flow cytometry analysis of surface markers demonstrated that BMSC expressed CD44, CD73, CD90, and CD105 and were negative for hematopoietic cell marks CD34, CD45, and HLA DR. White scale bar = 200 μm. Black scale bar = 100 μm.

**Supplementary Figure 3: Characterisation PDX PCa cells following mouse cell depletion**: PDX were enzymatically digested into a single cell suspension, characterises via flow cytometry, then depleted of mouse cells, and again characterised by flow cytometry. Prior to mouse cell depletion, most cell digests contained mostly mCD45 cells (histograms show unstained control cells (blue) and stained cells (red)). Post-depletion marker expressions are shown in the last four columns: mCD45, hCD44, EpCAM, hPSMA. (**a**) Larger diameter LuCaP35 cells were visible in the SSC/FSC plots. When depleted of mouse cells, LuCaP35 populations were rich in hCD44 (77.6%), EPCAM (90.8%) and PSMA (33.1%) (n=6 digested samples). (**b**) LuCaP141 cells could not be identified in SSC/FSC plots based on size alone. Depleted of mouse cells, enriched LuCaP141 cell populations contained an average of hCD44 (46.5%), EPCAM (70.9%), hPSMA (62.8%) human cells (n=5 digested samples). (**c**) BM18 cells were the smallest in size and not obvious on the SSC/FSC plots. Depleted of mouse cells, enriched BM18 cell populations contained an average of hCD44 (53.1%), EPCAM (92.6%), hPSMA (75.6%) human cells (n=3 digested samples). Bars equal averages, error bars equal one standard deviation.

**Supplementary Figure 4.** PDX-derived cells did not increase in number during 2D monolayer culture, and over time the viability of the remaining cells declined.


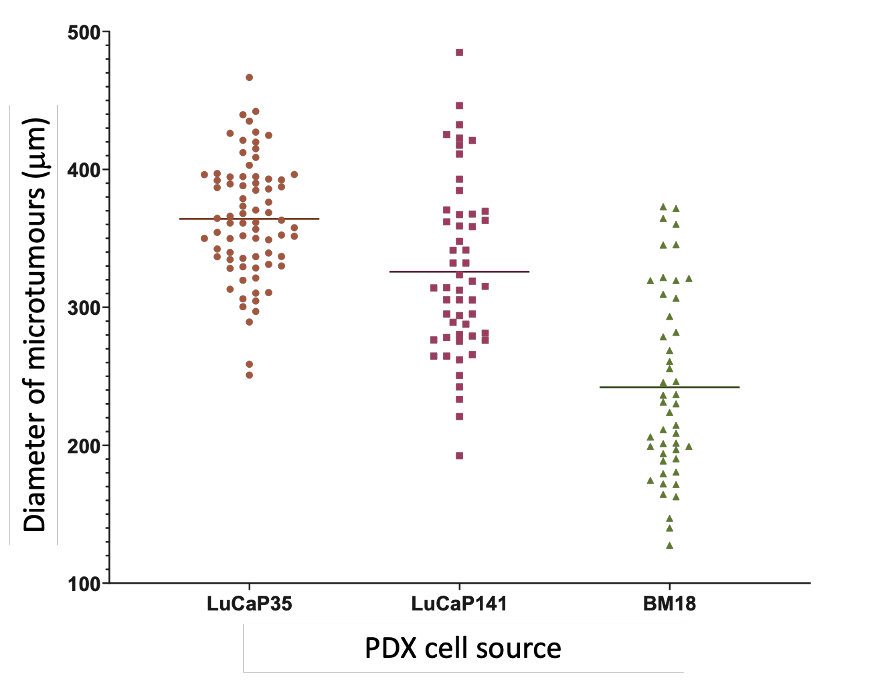


**Supplementary Figure 5:** Characterisation of microtumour size following overnight culture, and prior to implantation into animals. Each symbol represents a single microtumour. LuCaP35 microtumours were 364±42 µm in diameter (n = 75). LuCaP141 microtumours were 325±63 µm in diameter (n = 52). BM18 microtumours were 242±68 µm in diameter (n = 46).


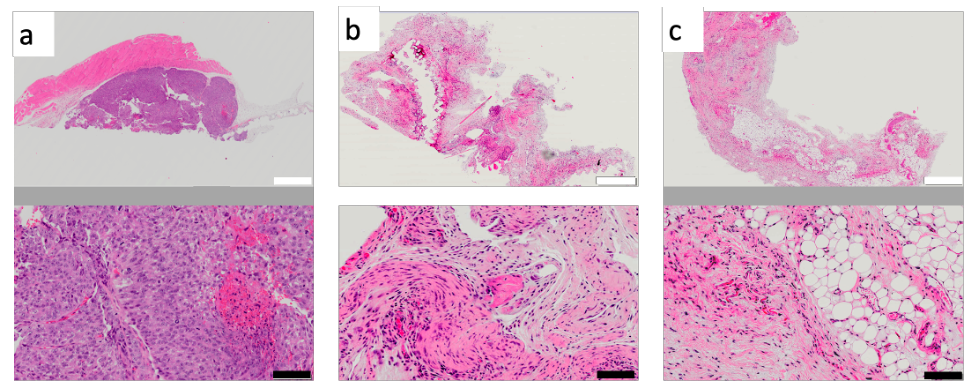


**Supplementary Figure 6.** H&E staining of micro-tumour tissue that overgrew the mesh. (a) LuCaP35, (b) LuCaP141, and (c) BM18. White scale bars = 1 mm, black scale bars = 200.

**Supplementary Table 1:** HCM Formulation adapted from Gao *et al*. ^10^. Medium was prepared fresh for each exchange or seeding.

| **Additive Agent** | **Concentration** | **Supplier** |
| --- | --- | --- |
| EGF | 50 ng/mL | Peprotech |
| FGF10 | 10 ng/mL | Peprotech |
| FGF2 | 1 ng/mL | Peprotech |
| Nicotinamide | 10 mM | Sigma |
| B27 Additive | 1x | Invitrogen |
| N-Acetyl-L-cysteine | 1.25 mM | Sigma-Aldrich |
| Glutamax | 2 mM | Invitrogen |
| HEPES | 10 mM | Sigma-Aldrich |
| SB202190 | 10 µM | Santa Cruz Biotechnology |
| Y-27632 | 10 µM | Selleck Chemical |
| A83-01 | 0.5 µM | abcam |
| R-spondin 1* | 1 ug/mL | Peprotech |
| Noggin* | 100ng/mL | Peprotech |
| Primocin | 1% v/v | InvivoGen |
| Dihydrotestosterone (DHT) | 0.1nM (CRPC)  1nM (hormone sensitive) | Sigma-Aldrich |

*In previous publications, these are sourced from conditioned medium generated by Lenti-X 293T cells (Clontech) and utilised respectively at 5% (R-spondin) and 10% (Noggin) v/v ^10^. Conditioned medium was sourced from Dr. Ian Vela in early experimentation with HCM. Due to limited availability, these stocks were replenished from commercial venders for the majority of experiments described here.
